# Fast kinetics of environmentally induced α-synuclein aggregation mediated by structural alteration in NAC region and result in structure dependent cytotoxicity

**DOI:** 10.1038/s41598-020-75361-6

**Published:** 2020-10-27

**Authors:** Tulika Srivastava, Ritu Raj, Amit Dubey, Dinesh Kumar, Rajnish K. Chaturvedi, Sandeep K. Sharma, Smriti Priya

**Affiliations:** 1 Systems Toxicology and Health Risk Assessment Group, CSIR-Indian Institute of Toxicology Research, Vishvigyan Bhawan 31, Mahatma Gandhi Marg, Lucknow, Uttar Pradesh 226001 India; 2 Academy of Scientific and Innovative Research (AcSIR), Ghaziabad, 201002 India; 3 Centre for BioMedical Research, Sanjay Gandhi Post Graduate Institute of Medical Sciences Campus, Raebareli Road, Lucknow, Uttar Pradesh 226014 India; 4 Department of Biotechnology, Majhighariani Institute of Science and Technology, Rayagada, Orissa 765017 India; 5 Food, Drug & Chemical Toxicology Group, CSIR-Indian Institute of Toxicology Research, Lucknow, Uttar Pradesh 226001 India

**Keywords:** Biochemistry, Chemical biology, Structural biology, Diseases, Risk factors

## Abstract

Aggregation of α-synuclein (α-syn) is associated with the manifestation of various pathogenic synucleinopathies, including Parkinson’s disease attributed to both genetic and environmental stress factors. The initial events triggering α-syn aggregation and disease initiation due to environmental stress factors are still largely unknown. Here, to understand the mechanism of misfolding and aggregation initiation, we induced α-syn aggregation with rotenone, an established chemical inducer of PD like symptoms. We found that rotenone accelerates the formation of structurally distinct oligomers and fibrils that act as templates and increase the formation of conformers capable of spreading to the neighboring neuronal cells. Molecular dynamics simulations and NMR studies revealed the involvement of NAC region and formation of helical conformations resulting in structural variations in oligomers and fibrils. These structural variations affect the cytotoxic potential of oligomers and fibrils, where, the beta sheet rich oligomers and fibrils alter the membrane potential of neuronal cells and lead to early apoptosis. Our results describe the initial mechanistic events in pathogenic protein aggregation, where initial structural alterations in response to external stress factors dictate the toxicity of resulting conformers. This information will further provide insights in the understanding of protein aggregation, disease progression and pathogenesis.

## Introduction

Cellular homeostasis is maintained by proteins folded into a specific, three-dimensional native conformation determined by their distinctive amino acid sequence^1,2^. The favored native state of a polypeptide is defined unless stimulated by external stress factors that result in protein misfolding and aggregation^3^. Stress factors such as mutations, errors in protein biogenesis, environmental stress and aging exhaust the cellular protein quality-control system and direct the affected proteins to misfold^4^. The misfolded proteins lose their biological activity and show a high propensity to aggregate. The protein aggregates may further interact with other cellular components leading to the impairment of vital cellular processes implicated in many protein aggregation related disorders^5^. The diagnostic feature common to protein misfolding disorders is the deposition of these insoluble protein aggregates and amyloid fibrils^6^. Parkinson’s disease (PD) belongs to this larger category of neurodegenerative protein misfolding disorders, where α-synuclein (α-syn) aggregate and accumulate to form pathological inclusions with neurofibrillary tangles of Lewy bodies^7,8^. Usually, the mechanism of aggregation for amyloids involves two unimolecular structural conversion steps, from the disordered to more compact oligomers and then to fibrils, that can further elongate by the addition of monomers^9^. In case of α-syn aggregation, the primary nucleation of monomers occur at very slow rates and is less studied in literature, whereas the secondary nucleation of fibril elongation is well understood; and reported to be influenced by pH^10^, lipid membrane and its components^11,12^, hydrophobic polymers^13^ , small molecules^14,15^, peptides^16^ and pesticides^17^. Further, the effect of these environmental inducers is not known on the primary nucleation of α-syn monomers, which may initiate aggregation as well. Lipid membranes have been reported to interact and trigger the conversion of α-syn from its soluble state into the aggregated state by enhancing the rate of primary nucleation and amyloid formation^18^.

The aggregation of α-syn in various synucleinopathies including PD is attributed to both environmental stress factors and genetic mutations^19^. Among environmental factors, pesticides (MPTP, paraquat and rotenone) are epidemiologically linked with the high risk of PD and their exposure in early years of life enhances probability to develop PD with aging^20^. Also, pesticides have been used to develop animal models for PD^21–23^, where they may act as neurotoxicants and affect the key pathogenetic processes, including the degeneration of nigrostriatal dopaminergic neurons through mitochondrial inhibition and accumulation of pathological α-syn aggregates^24,25^. The role of environmental factors such as pesticides in the initiation and progression of protein aggregation disorders is still unclear due to the lack of quantitative investigations in aggregates initiation at the primary nucleation stage. Owing to the complexity of aggregation mechanism of α-syn and involvement of external factors, it has been challenging to develop effective strategies to suppress the formation of α-syn aggregates and their toxicity. Here, we investigated the initial events of α-syn aggregation in the presence of rotenone and found that upon binding to rotenone, α-syn undergoes overall structural compaction, shows accelerated oligomerization rates and results in the formation of highly cytotoxic oligomeric and fibrillar species. This study strengthens the understanding of initial mechanistic events leading to the aggregation of pathogenic proteins in response to environmental risk factors significant in neurodegenerative diseases.

## Results

### Structural characterization of rotenone-induced α-syn oligomers and fibrils

**Figure 1:**
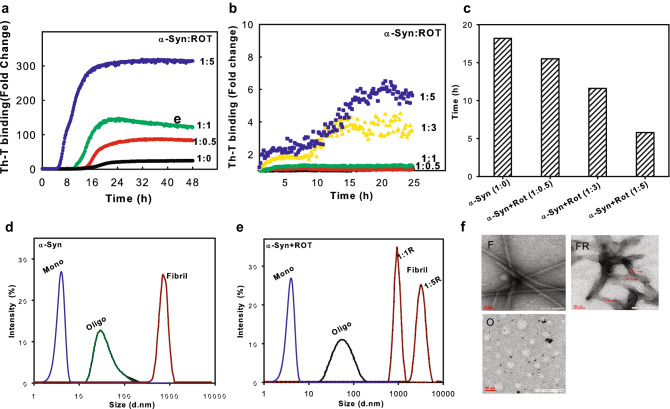
Rotenone accelerates α-syn aggregation by reducing lag phase. Kinetics of α-syn fibrilization (**a**) and oligomerization (**b**) induced by varying concentration of rotenone(ROT) (as shown α-syn:ROT ratios). The fluorescence intensity of thioflavin-T upon binding to misfolded and aggregated α-syn was measured for kinetic analysis. The start of exponential rise in ThT binding counted as lag phase from the fibril kinetics curves of 1a (**c**). Dynamic light scattering (DLS) for hydrodynamic radii analysis of α-syn monomer, oligomers and fibrils in the absence (α-syn, **d**) and presence of rotenone (α-syn + ROT, **e**). Structural morphology of the oligomers and fibrils (O, F) and rotenone induced fibrils at 1:5 ratio of α-syn:ROT (FR) was analyzed by transmission electron microscopy (TEM) (**f**). The fibrils and oligomers were prepared at agitating and static conditions respectively as described in material and methods. (Supplementary information: Table S1, Fig. S1a–c).

The standard aggregation kinetics of α-syn was performed using Thioflavin-T (Th-T) binding and fluorescence measurement. The oligomerization of α-syn was performed at static conditions, whereas the α-syn fibrilization proceeded with continuous shaking at 600 rpm for 48 h (See “Materials and methods”)^26^. Rotenone accelerated the aggregation of α-syn under both static and agitating conditions, where α-syn monomers ensemble into fibrils at a nearly 50-fold faster rate in the presence of rotenone as compared to α-syn alone and the lag phase of aggregation was significantly reduced (Fig. 1a–c). The Th-T binding kinetics suggests that rotenone enhanced the yield of α-syn fibrilization in a dose-dependent manner (Fig. 1a). Similarly, during the oligomerization of α-syn, faster rates and higher yields were observed in the presence of rotenone (Fig. 1b). Owing to the inherently transient nature of oligomeric intermediates, the net Th-T fluorescence of oligomers formed at static conditions is lower as compared to the fibrils. Rotenone significantly reduced the lag phase of fibril formation to 5 h as compared to α-syn alone 18.2 h for exponentially start of Th-T binding (Fig. 1c). Suggesting rotenone accelertaes aggregation kinetics of α-syn by reducing the lag. Further, the Th-T kinetics for both oligomers and fibrils follow a sub-linear behavior similar to that observed for other pathogenic proteins^27,28^ following a two-step Michaelis–Menten kinetics^29^.

Further, we characterized the rotenone-induced α-syn conformers for their size, structure and morphology. The oligomers and fibrils of α-syn formed were further analysed for morphology using transmission electron microscopy (TEM) (Fig. 1f). We observed that rotenone-induced fibrils are more complex and thicker with a width of 20–25 nm, as compared to the control α-syn fibrils that are frail, less complex and approximately 10–15 nm wide. Also, the rotenone-induced fibrils appear to be more branched and stacked. However, the morphological differences among rotenone-induced and uninduced oligomers could not be differentiated using TEM and AFM (Figs. 1f, S1c).

The approximate molecular weight and hydrodynamic radii of the fibrils and oligomers was characterized using size exclusion chromatography and dynamic light scattering (Figs. 1d,e, S1b). The distribution of the proportion of gyration radius versus the mass for α-syn is 5.7 nm which is consistent for monomeric α-syn as evident with dynamic light scattering. For oligomers and fibrils the gyration radii were observed between 50–100 nm and 800–5000 nm, respectively (Table S1). In the presence of rotenone, large fibrils of α-syn were formed, where the size of the oligomer was unaffected as analyzed by DLS (Fig. 1d,e). Further, with size exclusion chromatography, the oligomers appeared at approximately 8 ml, corresponding to a molecular weight of more than 500 kDa (Fig. S1b, Table S1). These techniques could not differentiate between rotenone-induced and uninduced oligomers, however, fibrils showed larger hydrodynamic radii. The dot blot analysis of various α-syn conformers using specific antibodies validated the purity of monomers and recognized oligomers and fibrils (Fig. S1a).

### Rotenone induces secondary structure variations in α-syn fibrils and oligomers

**Figure 2:**
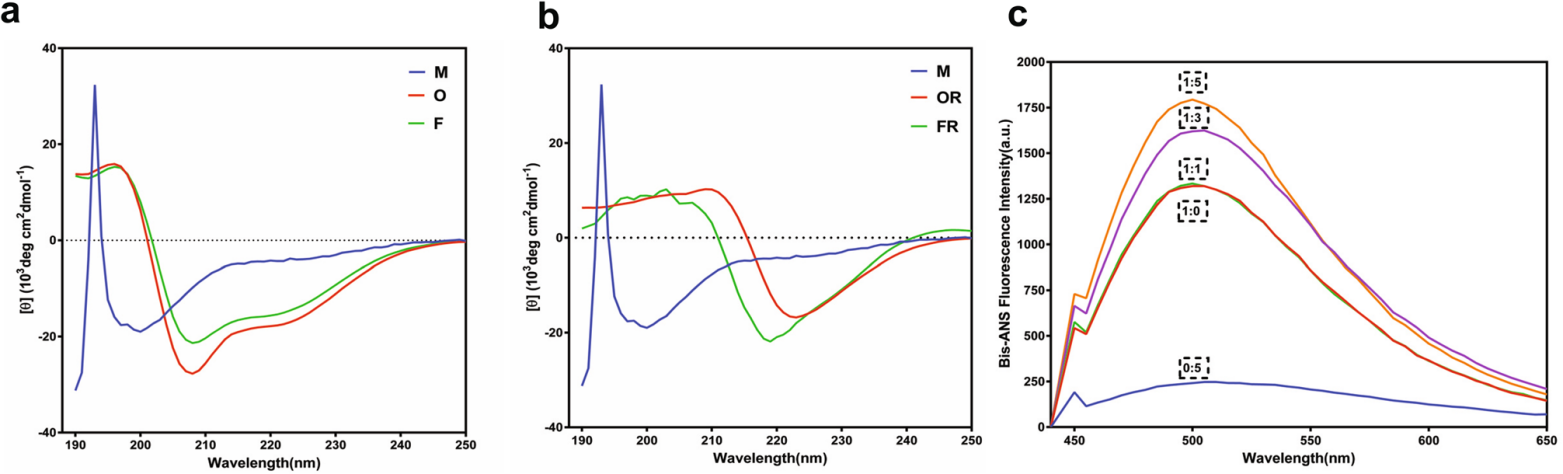
Rotenone induce β-sheet rich α-syn oligomers and fibrils. Far-UV spectra of α-syn monomers(M), oligomers(O) and fibrils(F) (**a**), rotenone induced olgimers (OR) an fibrils(FR) (**b**) shows characteristic changes in the secondary structure content of rotenone-induced α-syn oligomers. The hydrophobicity of α-syn in the presence and absence of rotenone measured using fluorescence emission of bis-ANS (**c**). (Supplementary information: Table S2a,b, Fig. S2).

During α-syn aggregation various intermediates or conformers formed are structurally different and alterations in the secondary structures can provide important information for their pathogenicity. Therefore, we measured the changes in the secondary structure of oligomers and fibrils induced by rotenone using far-UV circular-dichroism spectroscopy. The spectrum for monomeric α-syn shows minima at around 198 nm, consistent with the intrinsically disordered proteins (Fig. 2). In the absence of rotenone, the α-syn oligomers and fibrils showed minima at 208 and 222 nm (Fig. 2a), indicating the presence of α-helices, disordered and turns as calculated with BeStSel and K2D3. Interestingly, rotenone-induced α-syn oligomers have only one minima at 222 nm indicating the prevalence of β-sheet structures similarly, the rotenone-induced fibrils were also composed of only β-sheets (Fig. 2b). Quantification of secondary structure content using external software BeStSel (Beta structure selection) also validated the loss of α-helices in rotenone induced oligomers and fibrils (Table S2a). In the absence of rotenone, the α-syn oligomers and fibrils showed significant amount of α-helix structures along with other structures (Table S2). Thus, we found that rotenone-induced α-syn conformers are structurally distinct having α-helical alterations.

Natively unfolded or folded proteins do not undergo amyloid formation until high hydrophobicity, low net charge or other stress factors that increase their propensity to form β-sheet structures^30^. Probably, rotenone upon interactions with α-syn altered the initial decisive events of misfolding and triggered aggregation. Therefore, we analyzed the rotenone-induced hydrophobic environment in the vicinity of α-syn using bis-ANS, a non-polar fluorescent dye^31^. When incubated with bis-ANS, α-syn in the presence of increasing concentrations of rotenone marked an increase in the emission intensity as compared to α-syn or rotenone alone (Figs. 2c, S2). Notably, rotenone increased the hydrophobicity around α-syn that initiates distinct structural changes leading to faster misfolding as observed in CD and Th-T assays (Figs. 1, 2).

### Rotenone compacts α-syn structure with more α-helical transformations

The secondary structure alterations observed by CD were further analyzed using molecular dynamic simulations. Here, we explored the apparent role of α-helices and β-sheet in the aggregation initiation of α-syn and the effect of rotenone, if any, on the structural organization of conformers. Several structures of α-syn have been determined using X-ray, NMR and electron microscopy, yet, most of the structures are available for small fragments of α-syn. Merely three NMR structures exist for full-length α-syn, where 1XQ8, 2KKW are micelle-bound and 2N0A is for pathogenic fibrils. Here, we used 1XQ8 structure as it covers the full-length monomeric α-syn^32–34^.

Docking confirmed that rotenone binds to the aggregation-prone NAC region of α-syn (Fig. S3)^35^. Flexible docking approach was used to validate the potential binding region further and to generate accurate α-syn-rotenone complexes as starting structures for MD simulations. Using Desmond module of Schrodinger, molecular dynamic simulations for α-syn and α-syn-rotenone complex were performed for initial 20 ns as described in material and methods.

**Figure 3:**
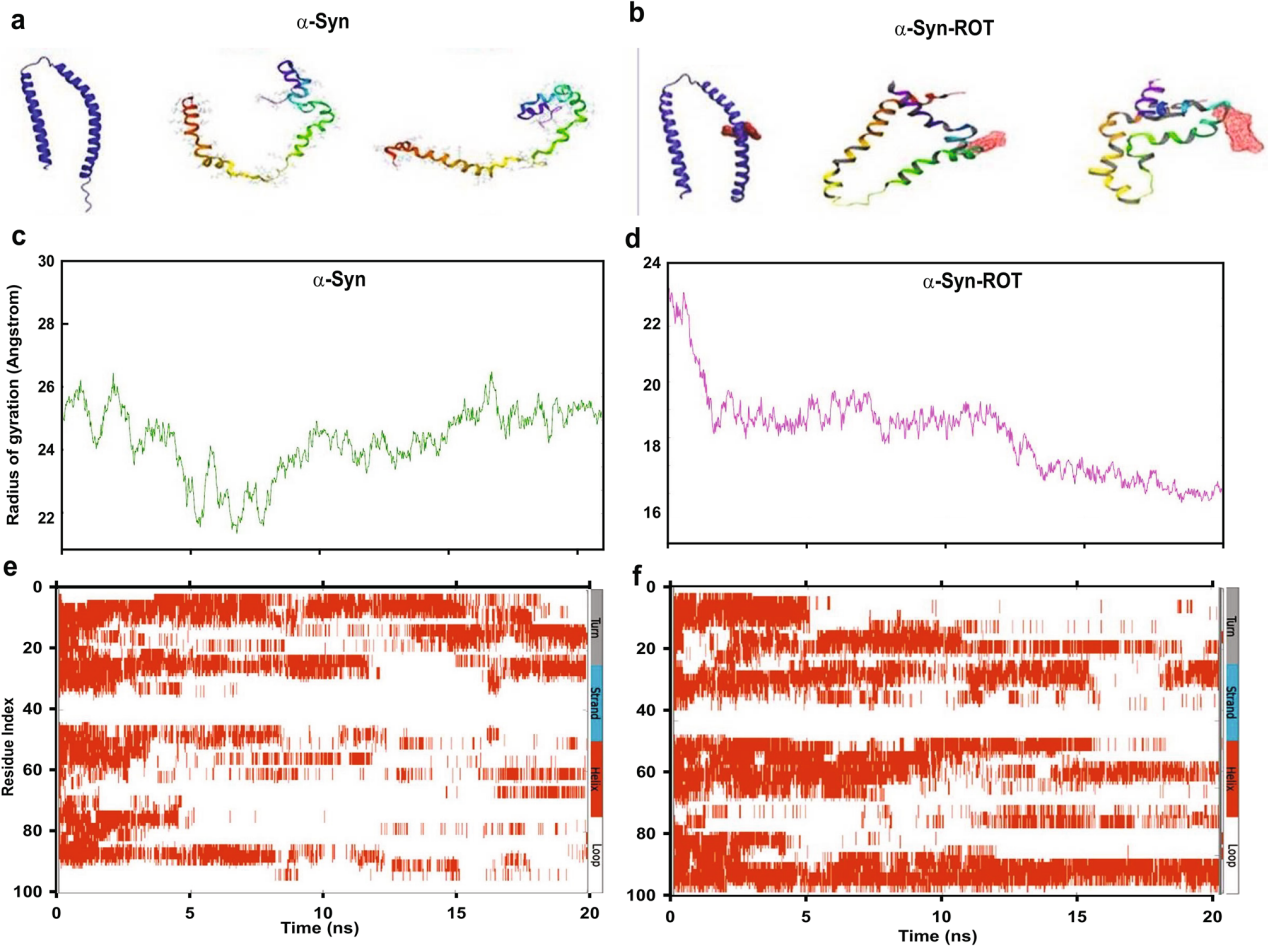
Rotenone binds to NAC region of α-syn causing structural compactness. Conformers of α-syn in the absence and presence of rotenone at 0, 10 and 20 ns of MD run (**a**). α-Syn retains its “candy-cane” structure, whereas in the presence of rotenone, α-syn structure compacts as measured by the radius of gyration (*R*_*g*_*)*. Compactness of α-syn structure *Rg* in the presence (**b**) and absence of rotenone (**d**). The structural changes in simulation time of 20 ns predicted using Database of secondary structure prediction (DSSP) plots of α-syn alone (**c)** and bound to rotenone (**e**Supplementary information: Fig. S3a–c).

After 20 ns run, α-syn was more disordered; however, rotenone bound α-syn had attained a comparatively compact structure (Fig. 3a,b). The radius of gyration, *R*_*g*_ measures protein compactness and for a stably folded protein maintains a relatively steady value. Loosely packed α-helices have a high radius of gyration whereas, low *R*_*g*_ values are characteristic of α/β-structures of the proteins. Rotenone bound α-syn showed decreasing R_g_ value indicating the probability of compact structure or β-sheets formation (Fig. 3c). For α-syn alone, fluctuations in *R*_*g*_ value suggests an unfolding stage for the protein (Fig. 3d).

Further secondary structure component analysis by DSSP^11^ revealed overall structural transformations in α-syn upon rotenone binding (Fig. 3e). The α-helical content in the α-syn-rotenone complex was 38.75% more as compared to α-syn alone (Fig. 3f) in the regions close to rotenone binding (Fig. S3b,c). Interestingly, we did not observe any β-sheets formation in both α-syn and α-syn-rotenone complexes during the simulation period. The rotenone-induced hydrophobic environment may be responsible for the initial helix formation in the vicinity of rotenone binding that later transforms α-syn into β-sheets rich oligomers (Fig. 2b) and contribute to the initiation of aggregation as observed with α-syn aggregation assays and CD experiments (Fig. 2a,b).

### Structural characterization of rotenone-α-syn interaction

**Figure 4:**
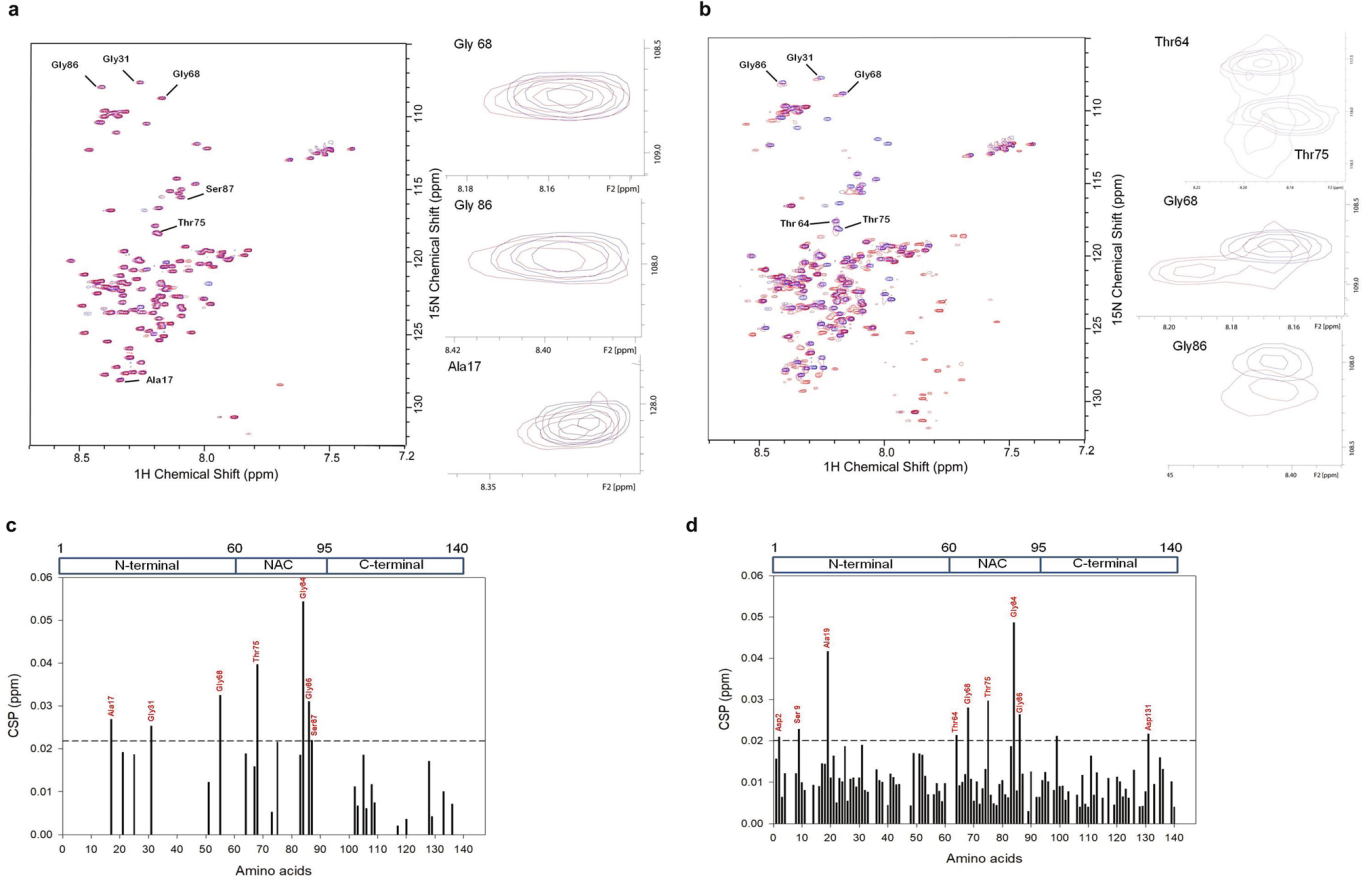
NAC region and N terminal of α-syn are involved in rotenone binding and aggregation. An overlay of the ^1^H–^15^N HSQC spectra of monomeric α-syn (blue) with preformed aggregated α-syn (red) (**a**), and rotenone (α-syn:ROT ~ 1:5) induced aggregated α-syn (red) (**b**). The aggregation was done at 37˚C and agitated condition for 48 h. Large chemical shifts in selected amino acids from N terminus and NAC regions are highlighted in spectra with arrows, and selected amino acid perturbations shown in panels at right. Chemical shift perturbation (CSP) plot of monomeric α-syn in comparison to aggregated α-syn in the absence (**c**) or presence of rotenone (**d**). CSP calculated as per emperical equation derived from delta H and delta N representing the chemical differences between 1H and 15 N respectively. (Supplementary information: Fig. S4).

To characterize the structural variations upon rotenone binding we conducted nuclear Magnetic Spectroscopy (NMR) of α-syn. The amide cross-peak assignments of ^1^H ^15^N HSQC spectrum of α-syn monomer prior to fibrillization established as per earlier studies^36^ (Fig. S4). NMR cannot determine the structure of oligomers and fibrils, therefore, we compared structural changes and amino acid perturbations from chemical shifts after 48 h aggregation. The amide chemical shift perturbations (amide CSPs) measured to probe the structural transitions due to rotenone binding by comparing monomer α-syn HSQC spectra with aggregated α-syn in the absence and presence of rotenone (Fig. 4a,b). Compared to monomeric α-syn, the subtle chemical shift perturbations were found in NAC region residues Gly68, Thr75, Gly84, Gly86, Ser87 and N terminus residues Ala17 and Gly31 in α-syn fibrils in the absence of rotenone (Fig. 4a,c). These shifts indicate structural alteration in fibrils. Further the α-syn fibrils induced by rotenone showed higher CSP (Fig. 4d) among NAC region residues (Thr 64, Gly68, Thr75, Glu83, Gly84, Gly86), N-terminal residues (Asp2, Ser 9, Ala19, Gly25, Gly31) as well as C-terminal residues (Asp131). These amplified and prominent peak shifts in rotenone-induced α-syn fibrils indicate that the conformational alterations may be induced by rotenone^36^.

### Rotenone induced α-syn oligomers and fibrils are cytotoxic for neuronal cells

Pathogenicity of *α*-syn is contributed by its prion-like propagation and the capacity to nucleate monomeric proteins into toxic conformers, where both of these pathogenic processes play a fundamental role in the initiation and progression of PD^37–39^.

**Figure 5:**
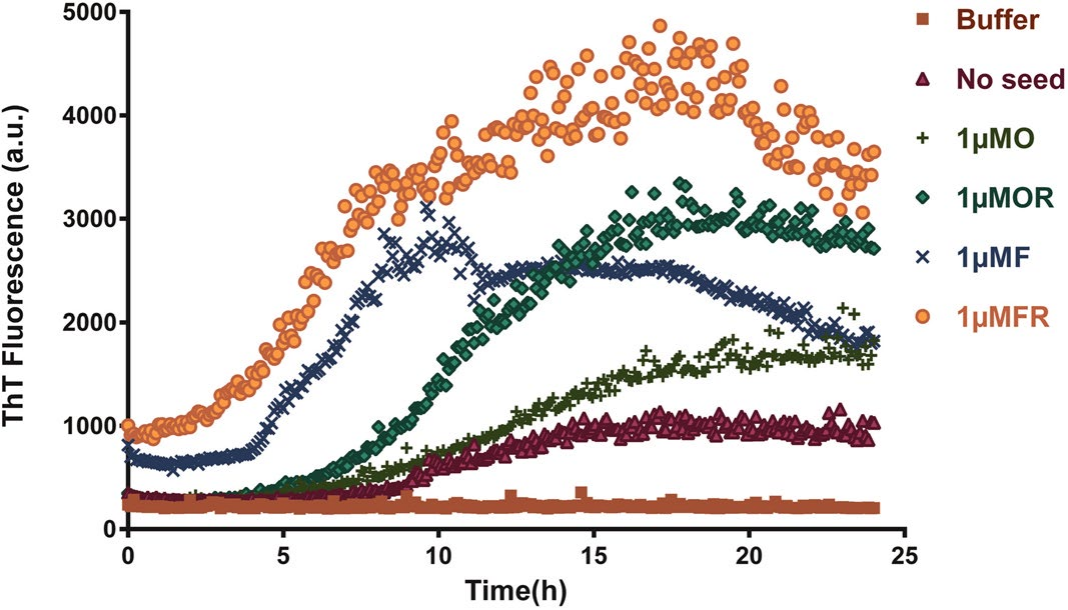
α-Syn oligomers and fibrils seed the aggregation of α-syn monomer. Preformed seeds of oligomers (O), rotenone induced oligomers (OR), fibrils (F) and rotenone induced fibrils (FR) (1 µM each) were added to monomeric α-syn (35 µM) in a controlled seeding experiment at 37 °C, linear shaking 600 rpm for 24 h. The resultant aggregation kinetics measured by Th-T fluorescence. No seeds were added in buffer. Both oligomers and fibrils were capable of enhancing primary nucleation rates of α-syn aggregation.

*In-vitro* seeding experiments suggest that oligomers and fibrils nucleate the aggregation of α-syn and diminish the lag phase significantly. Rotenone induced fibrils were more effective in reducing the lag phase and seeding *α*-syn aggregation (Fig. 5). The presence of seeds eliminates the need for primary nucleation and decreases the duration of the lag phase. We found that both oligomers and fibrils shift the equilibrium of a large amount of monomeric α-syn towards aggregation, contrary to earlier reports, where only insoluble fibrils were shown to nucleate the aggregation process^40^. In our study, rotenone-induced fibrils act more effectively in seeding as compared to the oligomers. The aggregation kinetics of α-syn also suggests preferential involvement of rotenone altered α-syn conformers in the nucleation and growth phases of aggregation.

To effectively serve as seeds for aggregation, exogenously added α-syn oligomers and fibrils must localize to the accessible cellular compartments as reported for fibrils of polyglutamine repeats and tau protein that are internalized by cells on the addition to the culture media^41^. Externally applied α-syn conformers have been shown to nucleate the polymerization of cytoplasmic α-syn and cause toxicity to cellular processes as well^42^.

**Figure 6:**
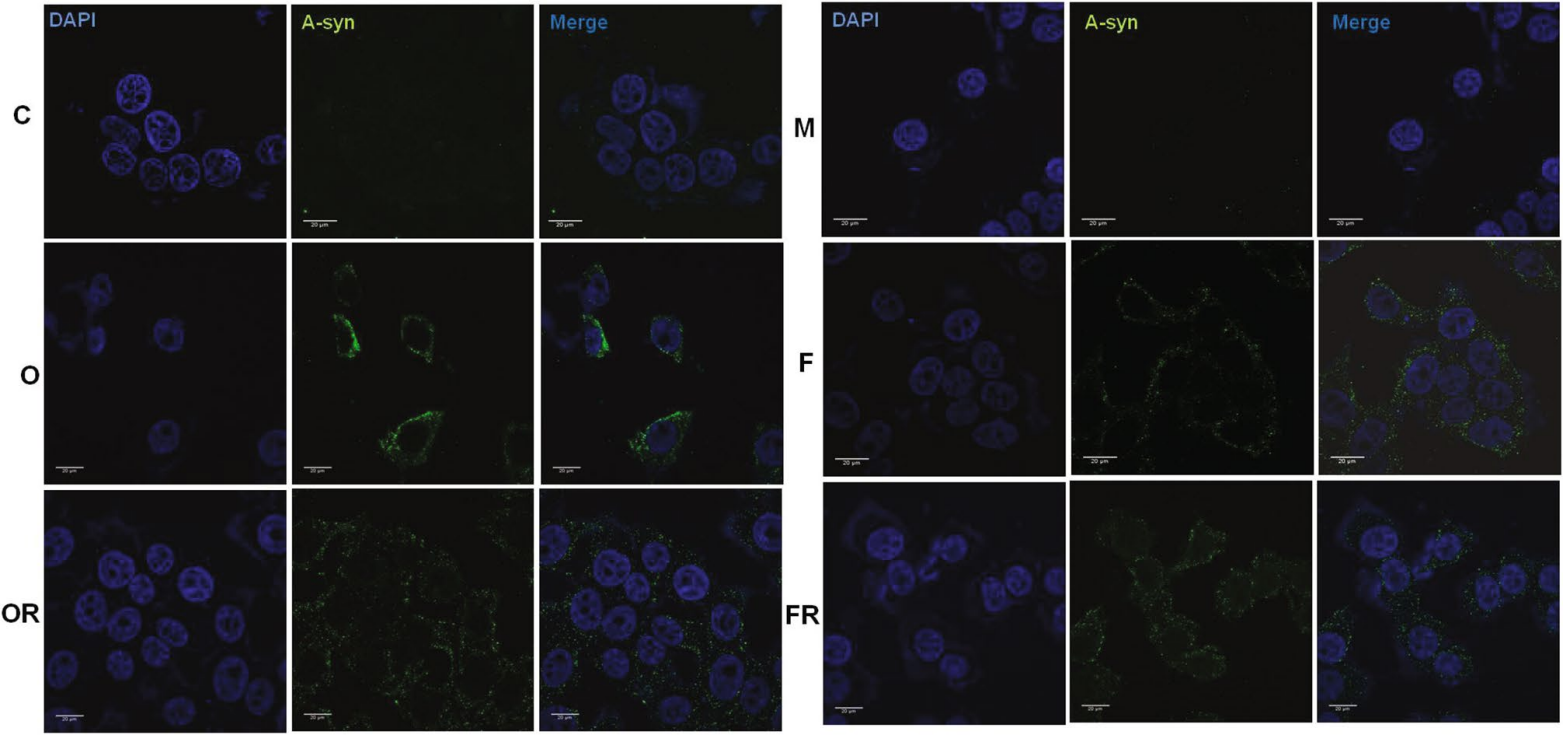
α-Syn oligomers and fibrils were internalized in human SH-SY5Y cells. SH-SY5Y cells were incubated with monomers (M), oligomers (O), rotenone-induced oligomers (OR), fibrils (F) and rotenone-induced fibrils (FR) (1 µM each) of α-syn using transfection reagent, buffer and controls for 24 h. For uptake studies cells were fixed with 4%PFA and subsequently stained for α-syn (alexa 488, green) and the nucleus was counterstained using DAPI (blue), scale bars = 25 μm.

For pathogenicity, we further investigated whether the oligomeric and fibrillar forms of α-syn could be efficiently introduced into the cytoplasm of SH-SY5Y neuronal cells. Mere addition of α-syn conformers in media did not result in any significant internalization into the cells. However, 24 h after using protein transfection reagent optimized for intracellular protein delivery, α-syn was detected in the cells as confirmed by confocal microscopy (Fig. 6). Transfected α-syn oligomers and fibrils were transiently distributed throughout the cytoplasm; however, fibrils were more spread (Fig. 6).

**Figure 7:**
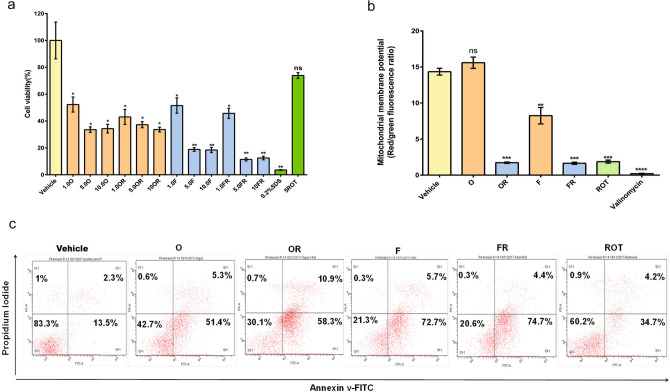
Rotenone induced α-syn oligomers and fibrils alter mitochondrial membrane potential and induce apoptosis. The cell viability on SH-SY5Y cells after 24 h treatment with α-syn aggregated species (O,OR,F,FR) at different concentrations(1–10 µM), rotenone (ROT, 5 µM) and SDS (0.2%) were assessed with MTT reduction assay (**a**) Percent cell viability is calculated to vehicle cell population normalized to 100%. Effect of α-syn aggregated species (O,OR,F,FR, 1 µM), rotenone (ROT, 5 µM) and valinomycin (0.3%) on mitochondrial membrane potential (ΔΨm) in SH-SY5Y cells measured by JC-1 staining (**b**). After 24 h treatment the cells were analyzed by flow cytometry and the quantitative ratio of red to green fluorescence is calculated. Late apoptosis induced by α-syn oligomers (O,OR, 1 µM) and fibrils (F,FR, 1 µM) measured by propidium iodide (PI) uptake (**c**). Flow cytometry analysis of apoptosis induced by the above mentioned treatments for48h after double labeling with PI and Annexin-V. Statistical significance was calculated by two tailed t-test and One way ANOVA analysis data expressed as means and ± SE (n = 3), *P < 0.05; **P < 0.01; ***P < 0.001;****P < 0.0001. (Supplementary information: Fig. S5a,b).

To understand the cytotoxicity mechanism, we assessed the oxidative stress, cell viability, apoptosis and mitochondrial membrane potential (Ψm) of SH-SY5Y cells on exposure to rotenone-induced α-syn oligomers and fibrils (Figs. 7, S5). Cytotoxic potential of α-syn conformers was investigated using 3-(4,5-dimethylthiazol-2-yl)-2,5-diphenyltetrazolium bromide (MTT) cell proliferation assay on the viability of human neuroblastoma SH-SY5Y cells. In cell proliferation assay all α-syn conformers were toxic as compared to the control (Fig. 7a) and significantly inhibited MTT reduction (p < 0.001) indicating loss in cell viability. α-Syn fibrils, both rotenone-induced and un-induced (FR, F) at 5 and 10 µM concentration were more toxic than oligomers in the cell proliferation assay (Fig. 7a). We further validated the cytotoxicity of the α-syn conformers by measuring the uptake of the vital dye propidium iodide (PI) as a marker for membrane disruption and apoptosis with flow cytometry quantification. α-Syn oligomers at a concentration of 1 µM caused 51.4% cells to undergo apoptosis, whereas rotenone-induced oligomers led to 58.3% apoptosis (Fig. 7c), and at the same concentration, rotenone-induced α-syn fibrils resulted in 72.7% apoptotic cell death (Fig. 7c). Both apoptosis and MTT reduction assay indicate that the rotenone-induced fibrils are more toxic than oligomers.

It is generally believed that apoptosis is caused by ROS generation^43^, the α-syn oligomers and fibrils did not induce any significant (p > 0.05) oxidative stress (Fig. S5a) in SH-SY5Y cells as measured by dichlorodihydrofluorescein diacetate (DCFDA).

As the depolarization of the inner membrane potential (Δψm) of mitochondria is considered as an early sign of apoptosis, tetraethylbenzimidazolylcarbocyanine iodide (JC1) dye assay was performed to ascertain the membrane damage potential of rotenone-induced α-syn oligomers and fibrils. In healthy cells, potential-dependent accumulation in mitochondria forms J-aggregates of JC-1 emitting red fluorescence, whereas, an increase in green fluorescence is observed upon depolarization of the mitochondrial membrane potential. Here we observed significant differences among rotenone-induced and other α-syn conformers. Rotenone induced oligomers and fibrils (OR, FR) remarkably increased the green fluorescence indicating a significant depolarization of the membrane (p < 0.0001) (Figs. 7b, S5b) In our studies the rotenone-induced α-syn conformers exhibited higher cytotoxicity as described above, the wildtype conformers were also toxic as expected and strengthened our hypothesis that structural distinction of conformers translates to pathogenicity. The cell-based toxicity studies for α-syn oligomers and fibrils further suggest that cell death is mediated by biochemical modifications leading to apoptosis.

## Discussion

Native proteins have a tendency to remain in folded state under physiological conditions^44^, however, environmental stress increases their probability for aggregation. The chameleon nature of α-syn also leads to the formation of different polymorphs with distinct toxicological profiles depending on the environment^25^ and exposure of pesticides^17,21,25^, metals^25^, lipids^11^, tetrafluoroethylene (TFE)^45^ and sodium dodecyl sulfate (SDS)^12^ have been reported to induce α-syn aggregation. However, what triggers the aggregation and the role of chemicals/pesticides in initiating protein misfolding is not yet known. In the present study, we explored the detailed mechanism involved in chemically (rotenone) induced α-syn aggregation and the pathogenicity of resulting conformers. Pesticides like rotenone has been successfully used to establish models of Parkinson’s disease in rats^21^ where the mechanism of chemical/pesticide induced PD is mainly attributed to oxidative stress^46^ and α-syn aggregation^25,47^.

We found that rotenone influence the initial α-syn aggregation via the formation of structurally distinct oligomeric intermediates. The lag phase in protein aggregation represents the primary nucleation step, which is slow due to high energy barriers for aggregation^10,48^ rotenone reduced the lag phase of α-syn aggregation by three-fold in comparision to α-syn alone (Fig. 1a–c)^28,49^. The resultant fibrils are rich in β-sheets and resemble previously reported aggregates formed by α-syn alone^50^, or induced by detergents and lipids^12,51^ and linked with PD initiation and progression^45^.

Reduction in lag phase and faster aggregation kinetics indicates the involvement of rotenone in either lowering energy barriers for aggregation or remodeling of the α-syn conformers. As evident from electron microscopy, rotenone-induced fibrils are relatively thicker, stacked and branched (Fig. 1f, Table S1) representing a higher order of aggregation complementing the ThT fluorescence of fibrils (Fig. 1a). Early saturation of Th-T fluorescence at the elongation phase also represents rapidly depleting monomer concentration.

We found that oligomers and fibrils have distinct secondary structural arrangements in the presence of rotenone (Fig. 2a,b). The rotenone-induced fibrils and oligomers showed presence of parallel and anti-parallel β-sheets, however, they lacked α-helices and disordered regions as found in monomers (Table S2). α-Syn oligomers formed in the absence of rotenone showed the presence of some α-helices and disordered regions as observed in membrane and micelle bound α-syn or slow aggregation by small molecule inhibitors^52^. In α-syn fibrils, the helix and disordered structural forms transform subsequently in to β-sheets. Slow aggregation or the presence of anti-aggregation compounds lead to the formation of fibrils with a significant amount of α-helices among pathogenic proteins. The helix-rich intermediate was mostly oligomeric and suggested to be an on-pathway intermediate of pathogenic aggregation. In α-synuclein aggregation, the N-terminal regions have a high propensity to acquire helical structure, that might mediate the slow formation of helix-rich intermediate through intermolecular interactions^52,53^.

Our results show that rotenone-induced oligomers and fibrils have an increased propensity to adopt β-sheet structures (Fig. 2) in concurrence to the higher Th-T fluorescence (Fig. 1a,b) and faster aggregation kinetics.

The natively unfolded state of α-syn is favored by relatively low hydrophobicity and high net charge^35,54^. We found that rotenone caused alterations in the environment and increased the hydrophobicity (Figs. 2c, S2), thus inducing misfolding favorable conditions and binds to the aggregation-prone region of α-syn (Fig. S3a). The presence of environmental factors, such as pesticide exposure, metals or stress can change the physiological conditions to more aggregation-prone conditions with enhanced hydrophobicity^13^ or net charge^30,35^ as observed in other protein aggregation studies. The hydrophobic environment may change the secondary structure of α-syn and alter the initial aggregation kinetics in the presence of rotenone. Further, for understanding the initial rot-α-syn interactions we did molecular dynamic simulations and NMR studies. We found that rotenone binds to aggregation-prone region of α-syn resulting in distinct structural reorganizational changes in α-helices. A 20 ns MD simulation shows that rotenone binding caused the formation of more α-helices (Figs. 3c, S3). Thus, the hydrophobic environment governs the initiation of aggregation pathway at the primary nucleation step, turns α-syn into structurally distinct, more compact and helical, which later catalyzes faster aggregation kinetics leading to the formation of β-sheet rich oligomers and fibrils. The α-helical conformations are reported to be directly involved in initial aggregation steps of α-syn and mediate the transition of unstructured monomers into β-sheet rich fibrils^45,55^.

The comparison of α-syn HSQC monomer spectra with 48 h aggregated spectra revealed prominent structural changes in the presence of rotenone. Aggregated α-syn after 48 h showed subtle chemical shifts in aggregation-prone non-amyloid-β component (NAC) region and N-terminal amino acids (Fig. 4a). These structural changes are in occurrence with previous investigations where the hydrophobic core of NAC region (residues 61–95) initiates the early assembly of α-syn and aggregation^56^. In rotenone-induced α-syn aggregation (Fig. 4b) we observed major structural changes in NMR spectra however, the prominent chemical shifts were amplified in NAC hydrophobic core region and N terminal amino acids (Fig. 4d). Rotenone amplified the misfolding events by providing a favorable environment and did not bring new structural alterations. The NAC region has been targeted for designing potent inhibitors of aggregation, but the direct interaction of inhibitors with the NAC region may not be necessary to inhibit pre-amyloid β-oligomer formation; there may be other non-covalent interactions leading to misfolding and cytotoxicity^57^. Another high throughput screening of compounds that possibly inhibit α-syn aggregation^58^, showed the majority of compounds interact with monomeric α-syn. Our findings of α-syn aggregation initiation and structural changes can be important for designing new inhibitor approaches effective in preventing aggregation.

The nature of the intermediate misfolded state preceding aggregation is a deciding factor for further events in the formation of conformers with altered affinities and distinct structural features known as “seeds” or “strains”. The physiological relevance of structural heterogeneity among α-syn strains is reflected in their seeding potential and toxicity mechanisms^26,59^. The α-syn strains from MSA and PD patients showed different morphology, biochemical properties and seeding capacities for α-syn monomers in cell systems^60^. In our study as well the α-syn seeds formed in the presence of rotenone showed higher seeding propensities and shifted the equilibrium of monomeric α-syn towards aggregation by reducing the primary nucleation step (Fig. 5). Further the structural differentiation translated into selective cellular toxicities. α-Syn oligomers and fibrils are more toxic than rotenone as they adopt the different mechanisms of toxicity rather than mitochondrial dysfunction or ROS generation (Figs. 7, S5a). The oligomers remain localized in the cells (Fig. 6) as reported earlier^61^ and we found that oligomers and fibrils exhibit localized toxicity as confirmed with membrane-binding potential (Figs. 7, S5) which was significantly higher for rotenone-induced conformers. Early apoptosis and compromised cell viability was independent of the nature of oligomers and fibrils, their structural heterogeneity or the presence of rotenone (Fig. 7c) and these conditions exhibited similar toxicity for cell viability/apoptosis. Here, we show that differences in the intrinsic architecture of oligomers and fibrils reflect their ability to propagate and bind to membranes as reported earlier for structurally distinct α-syn polymorphs (fibrils and ribbons) having higher toxicity and the capability to nucleate seeded aggregation^39^.

**Figure 8:**
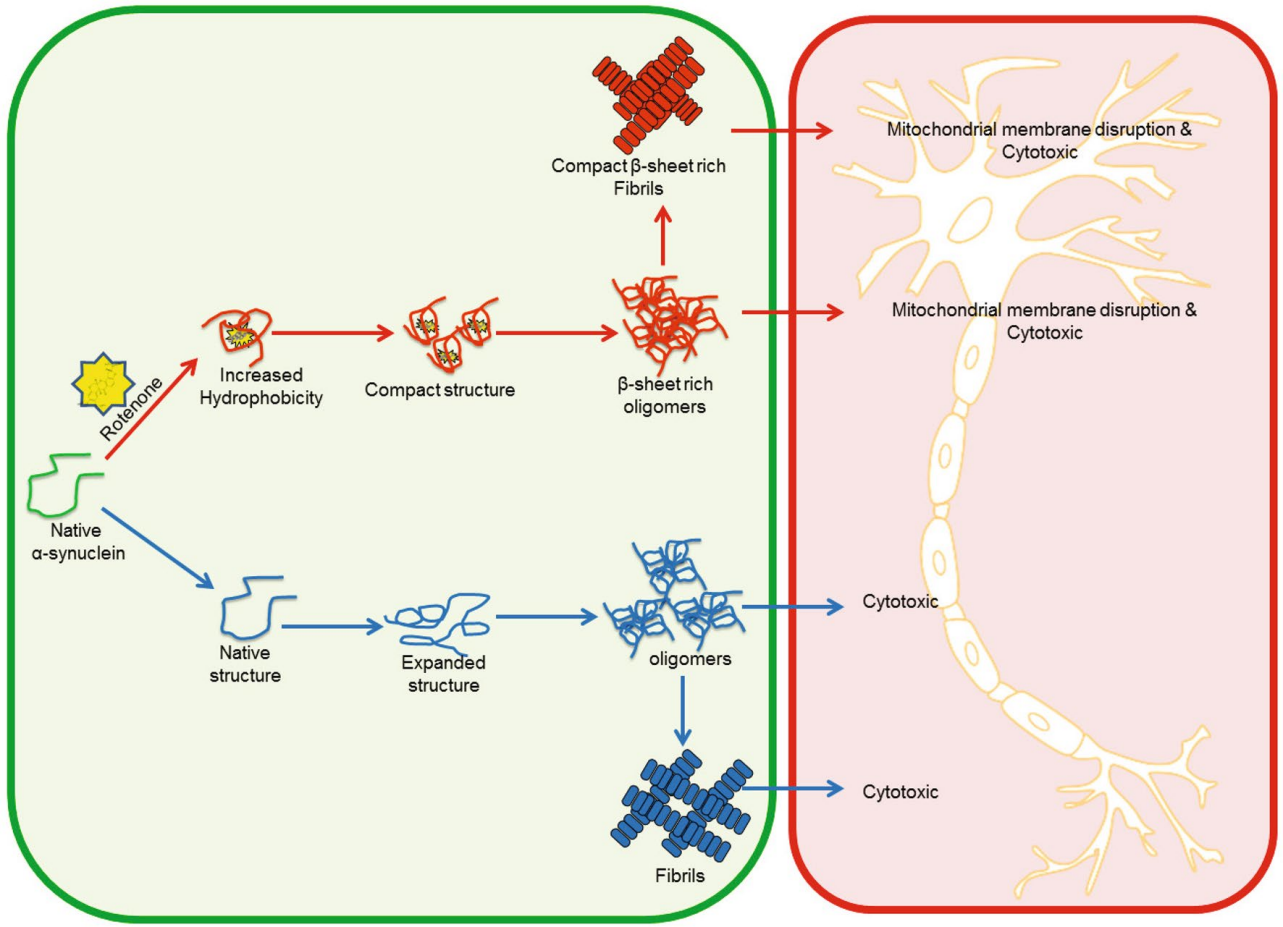
Schematic representation of rotenone induced α-syn aggregation and cytotoxicity mechanism. Rotenone-induced pathway represented as red arrows in comparison to normal α-syn aggregation (blue arrows).

To summarize, the initiation of α-syn aggregation begins with rotenone-α-syn interactions, which accelerates the formation of structurally distinct oligomers and fibrils (Fig. 8). These oligomers and fibrils act as templates to promote the misfolding and aggregation which subsequently results in an increased number of aggregates that can spread to the neighboring cells in PD pathogenesis^62^. As the seeds of pathogenic proteins are highly stable and reserve their toxicity and prion-like properties in structural folds that defines their inherent pathogenic nature in the onset of diseases. The seeds of amyloid β (Aβ) preserved for more than five decades in growth hormones were found to be active and capable of inducing Alzheimer’s disease in animals^63^. Similarly, environmental exposure may also induce misfolding in α-syn to form seeds that may remain dormant till further exposure, favorable genetic conditions or aging for disease progression. Given that structurally variable α-syn conformers are linked to diverse etiologies of synucleinopathies, this study explains the mechanisms involved in the initiation of PD upon environmental exposure.

## Materials and methods

In this study, we investigated the α-syn aggregation process in the presence of rotenone, a pesticide, using various biophysical and imaging techniques including Th-T fluorescence assays, ANS binding, CD spectroscopy, dynamic light scattering and TEM. The toxicity of α-syn conformers was studied on neuronal SH-SY5Y cells using MTT assay, Annexin-V apoptosis assay, ROS detection assay and mitochondrial membrane potential assessment. The detailed description of methods is provided in the Supplementary information.

## Supplementary information

Supplementary Information.
