# Supplementary material for: Fast kinetics of environmentally induced α-synuclein aggregation mediated by structural alteration in NAC region and result in structure dependent cytotoxicity
Source: Sci Rep. 2020 Oct 27;10:18412. doi: 10.1038/s41598-020-75361-6 (PMC7591854; doi:10.1038/s41598-020-75361-6)
Supplement: Supplementary file 1 — Supplementary Information. [file 41598_2020_75361_MOESM1_ESM.docx]

**Supplementary Information**

Fast kinetics of environmentally induced α-synuclein aggregation mediated by structural alteration in NAC region and result in structure dependent cytotoxicity

Tulika Srivastava^1,2,^  Ritu Raj^3^, Amit Dubey^1,4,^, Dinesh Kumar^3^, Rajnish K Chaturvedi^1,2^, Sandeep K. Sharma^2,5^, Smriti Priya*^1,2^

^1^Systems Toxicology and Health Risk Assessment Group, CSIR-Indian Institute of Toxicology Research, Lucknow 226001, Uttar Pradesh, India.

^2^Academy of Scientific and Innovative Research (AcSIR), Ghaziabad- 201002, India

^3^Centre for BioMedical Research, Sanjay Gandhi Post Graduate Institute of Medical Sciences Campus, Raebareli Road, Lucknow 226014 , Uttar Pradesh, India.

^4^Department of Biotechnology, Majhighariani Institute of Science and Technology, Rayagada 765017, Orissa, India

^5^Food, Drug & Chemical Toxicology Group, CSIR-Indian Institute of Toxicology Research, Lucknow 226001, Uttar Pradesh, India.

***Corresponding Author:**

Dr. Smriti Priya

Systems Toxicology and Health Risk Assessment Group

CSIR-Indian Institute of Toxicology Research

Vishvigyan Bhawan 31, Mahatma Gandhi Marg

Lucknow 226001

UP, India.

Email: [spriya@iitr.res.in](mailto:spriya@iitr.res.in), [smritipriya3@gmail.com](mailto:smritipriya3@gmail.com)

**α-Syn oligomers and fibrils preparation:**

WT α-syn plasmid (pT7-7 α-syn WT, a gift from Hilal Lashuel Lab) was transformed in BL21 (DE3) cells. The expression and purification of α-syn was done using anion exchange column (Q-sepharose 26/10 high performance, GE Healthcare, UK) and size exclusion column (Hiload 26/60 Superdex 75 preparative, GE Healthcare, UK) as previously described^[i]^ **.** The purified protein was lyophilized and stored at -80˚C.

**Oligomers:** α-Syn oligomers were prepared as previously described^[^[^26^](#_ENREF_23)^]^. Briefly, α-syn (70µM) was suspended in PBS buffer, pH 7.4, filtered through a 0.22μm filter and subsequently incubated at 37°C for 24h in stationary mode, without agitation in order to avoid acceleration of fibril formation ^[^[^26^](#_ENREF_23)^]^. For rotenone induced oligomers, monomeric α-syn was incubated in the presence of rotenone (molar ratios of α-syn:ROT; 1:0.5, 1:1, 1:3 and 1:5) followed by static incubation at 37°C for 24h. The small amount of fibrillar species were formed during this incubation period and that were removed by high speed centrifugation for 1h at 25000 rpm (Sorvall Lynx 6000, Thermo Fisher Scientific). The oligomers were stored at -80˚C after flash freezing in liquid N2 and utilized for experimental purposes within 3-4 days.

**Fibrils:** α-Syn fibrils were prepared by incubating monomeric α-syn (70µM) in phosphate buffer saline (PBS), pH-7.4, with a glass bead under constant linear agitation of 600 rpm at 37°C for 48h ^[^[^26^](#_ENREF_23)^]^. For rotenone induced fibrils, monomeric α-syn was incubated in the presence of rotenone (molar ratios of α-syn:ROT; 1:0.5, 1:1, 1:3 and 1:5) followed by linear shaking 600rpm at 37°C for 48h. The resulted fibrils were centrifuged (13200 rpm, 15 min), pellet was washed and re-suspended in equal amount of PBS. The protein concentration was determined by absorbance at 280nm in (Lambda 365 Perkin, spectrophotometer) and aliquot to store at -80˚C after flash freezing in liquid N2.

**α-syn aggregation kinetics measured by Th-T binding assay:**

The aggregation kinetics of oligomer and fibril formation was measured by Th-T fluorescence. Monomeric α-syn (100µM) with different concentration of rotenone(molar ratios of α-syn:ROT; 1:0.5, 1:1, 1:3 and 1:5) was suspended with ThT solution (20 μM) in PBS. The experiment was set in 96well plates with agitation and reading cycle of 300s (linear shaking, 1mm amplitude) to homogenize the samples ^[^[^29^](#_ENREF_10)^]^. Th-T fluorescence was measured using Infinite M200 PRO multimode plate reader (TECAN, Switzerland) at an excitation wavelength of 450nm and an emission wavelength of 480nm.

**Characterization of α-syn conformers:**

Dynamic light scattering: DLS measurements were performed at 25°C using a Zetasizer Nano-ZX (Malvern). The rotenone induced and un-induced α-syn monomers, oligomers and fibrils were further diluted in PBS (final concentration 10µM) and measured immediately. Scattering peaks less than 0.1 nm radius were ignored. Each α-syn sample was measured at-least five times in three experimental setups.

**Circular Dichroism spectroscopy:**

For CD spectra of α-syn monomers, oligomers and fibrils (35µM) were placed in 1mm path length quartz cell. The CD spectra were recorded from 260 to 190nm at 25°C using CD spectrophotometer (JASCO-1500, Japan). The final spectrum was obtained from average of three consecutive scans and subtracting buffer spectra according to manufactures protocol.

**Dot blot and western analysis:**

α-Syn monomers, oligomers and fibrils (35µM), in PBS, pH 7.4, were spotted on duplicate nitrocellulose membranes for dot blot analysis. The membranes were blocked in 5% BSA and probed with monomer specific anti α-syn (ab52168, Abcam), fibril specific syn211 (ab80627, Abcam) or oligomer specific A11 (AHB0052, Invitrogen) antibodies with appropriate dilutions. Following multiple washings with TBST, the membranes were incubated with HRP conjugated anti-mouse and anti-rabbit secondary antibody for 1h at room temperature and imaged with chemidoc (Amersham Imager 600 system, GE Healthcare Life Sciences, Pittsburgh, PA, USA).

**Size Exclusion Chromatography**:

α-Syn monomers (100µM), α-syn oligomers (100µM) and rotenone induced α-syn oligomers (35µM) were separated individually and analyzed by Superose^TM^ 6 Increase 10/300 GL gel filtration column attached to FPLC AKTA Pure 25L (GE Healthcare, UK). The protein was eluted isocratically at 0.5ml/min and the fractions collected were monitored by UV absorbance. The chromatograms were analyzed as per the manufacturer instructions and with standard marker proteins.

**Transmission Electron Microscopy (TEM):**

α-Syn fibrils and oligomers were diluted (5µM) with 1X-PBS.10µl of diluted samples were negative stained with uranyl formate and immediately placed on carbon coated copper grids. The specimens were examined through Gatan digital micrograph software under a FEI transmission electron microscope (Tecnai G2 Spirit, Netherlands) at 80 kV.

**Atomic Force Microscopy (AFM):**

The morphology of oligomers and rotenone induced oligomers observed using AFM (Solver Nano, NT-MDT Spectrum Instruments, Russia). Samples of oligomers and rotenone induced oligomers were spotted on a freshly cleaved mica sheet, incubated for 5−10 min, following with 2 times washing with double distilled water and air dried at room temperature. The imaging was performed in semi-contact mode with suitable cantilevers.

**Bis-ANS binding assay:**

The surface hydrophobicity of α-syn in the presence and absence of different concentrations of rotenone was measured with Bis-ANS, a specific hydrophobic probe. Monomeric α-syn (35µM) pre-incubated with rotenone (molar ratios of α-syn:ROT; 1:0, 1:1, 1:3 and 1:5) for 1h at 37˚c in PBS, followed by Bis-ANS (15μM) addition 10 mins prior to fluorescence reading. Fluorescence emission spectra were recorded from 450 to 650 nm after excitation at 420 nm using Infinite M200 PRO multimode plate reader (TECAN, Austria).

**Cell culture:**

SH-SY5Y (Human neuroblastoma, ECACC-94030304) cells were cultured in DMEM–F12 media supplemented with 10% fetal bovine serum (Life Technologies South American origin), 1% antibiotic-antimycotic (Life Technologies) at 37^0^C and 5% CO_2_ humidified atmosphere.

Cellular uptake of α-syn oligomers and fibrils by SH-SY5Y cells was analysed using fluorescence microscopy. SH-SY5Y cells were plated in 6 well culture plates till 60-70% confluency. Cells were transferred to serum-free media 1h before transfection. For each well, preformed α-syn fibrils or oligomers (1µM) were incubated with cationic-liposomal protein transfection reagent (15µL; Xfect^TM^ protein transfection reagent, Clontech) at room temperature for 30mins. For treatment, the cellular media was replaced with fresh media containing α-syn-protein transfection reagent. Equal amount of PBS was added to serve as control. After 6h treatment cells were washed twice to remove extracellular α-syn and transfected cells were maintained in media containing 10% FBS for 24h.

The α-syn transfected cells were further analysed for cellular uptake of α-syn species, cell viability, ROS generation, apoptosis and mitochondrial potential.

**MTT assay:**

The SH-SY5Y cells were seeded on a 96-well plate in 100µl fresh medium at 70-80% confluency. After 24h, the media was exchanged with fresh media containing preformed α-syn fibrils and oligomers (1, 5, 10µM). Controls samples, Rotenone (5µM, ROT) and SDS (0.2%), were prepared with the addition of identical volumes of buffer. After 24h of incubation, the cells were incubated for another 2-6h with 0.5 mg/ml MTT (Sigma) solution. Finally, the entire media was replaced by detergent reagent DMSO (100µl) in each well to solubilize the formed formazan crystals. The absorbance of formazan was measured at 570 nm in Infinite M200 PRO multimode plate reader (TECAN, Austria). P-values were calculated using two tailed t-test.

**Annexin V apoptosis assay:**

The SH-SY5Y cells were seeded in 6-well plate in standard cell culture media at 50-60% confluency and treated with preformed α-syn oligomers, fibrils (1µM) and rotenone (5µM, ROT) for 48h at 37˚C. After treatment the cells were trypsinized and spun down at 3000rpm for 1min. Then cell pellets were washed twice with PBS and re-suspended in 200µl of Binding buffer containing Annexin V-FITC (as per manufacturer instructions ApoDETECT Annexin V-FITC, 331200, Thermo Fisher Scientific). For propidium iodide (PI) staining, the cells were pelleted and re-dissolved in binding buffer containing 1µg/ml PI and immediately the acquisition and analysis was done using flow cytometer (FACS Canto^TM^ II, BD Biosciences, San Jose, CA, USA).

**Reactive oxygen species (ROS) detection assay:**

The SH-SY5Y cells were seeded in 96 well plate in standard cell culture media to obtain 70-80% confluency and treated with preformed α-syn oligomers or fibrils (0.2-2µM) for 6h at 37˚C. After treatment, the cells were washed and stained with 25µM redox-sensitive dye dichlorodihydrofluorescein diacetate (DCFDA) for 45 min at 37°C under dark conditions. DCFDA solution was removed after incubation time and fluorescence was measured at excitation/emission 485/535 nm in end point mode with Infinite M200 PRO multimode plate reader (TECAN, Austria).

**Mitochondrial membrane potential (MMP) assessment**:

The SH-SY5Y cells were seeded in 6-well plate in standard cell culture media to 50-60% confluency and treated with preformed α-syn oligomers, fibrils (1µM) for 24h at 37˚C. After treatment the cell pellet was re-suspended in fresh complete medium containing 5µg/ml of JC1 staining solution (as per manufactures protocol, CS0390 Sigma) and incubated for 20 minutes. Additional positive control of valinomycin (V3639, 0.3%) was added to the staining solution and incubated with other samples as per protocol. After treatment the cells were pelleted, washed with JC-1 staining buffer and re-suspended again in 500µl of the ice-cold JC-1 staining buffer for immediate acquisition and analysis of stained cells using flow cytometer (FACS Canto^TM^ II, BD Biosciences, San Jose, CA, USA).

**Immuno-fluorescence Studies**:

SH-SY5Y cells were seeded on glass cover slips at 70-80% confluency and transfected with preformed α-syn fibrils or oligomers (1µM) as described above. The cells treated with PBS were used as control. Following the treatment, cells were fixed in 4% formaldehyde for 10mins at room temperature and washed twice with 1X-PBST containing 0.3% Triton-X. Further cells were blocked with 3% bovine serum albumin (BSA) and subsequently incubated with Syn211 (ab80627, Abcam) and alexa 488 (ab150113 Abcam) for 2h at room temperature. After multiple washings with PBS, cells were mounted using vectashield mounting media conaiting DAPI (Vector laboratory, CA) and imaged at 60X resolution using appropriate filters for DAPI (excitation wavelength 340-380nm) and FITC (excitation wavelength 450-490nm) using confocal microscope (Leica TCS SPE, Germany).

**Molecular dynamic simulation of α-syn:**

The structure of the α-syn bounded with ligand (rotenone) in both N and C terminal complexes were selected for molecular dynamics simulation. Total three systems were generated for simulation, 1^st^ for alone protein, 2^nd^ for N-terminal bounded ligand and 3^rd^ for C-terminal bounded ligand. All three simulations were solvated in 4x4x4Å triclinic periodic box with SPC water molecules and neutralized with adding NaCl ions in absolute buffer conditions. All three complex were analyzed for stability and NAC region bound ligand was selected for further simulations. The structures were energy minimized for 2000 steps of steepest descent algorithm followed with 5000 conjugate gradient algorithm with 120.0kcal/mol threshold energy. MD simulations were carried out with Desmond v8.8 academic version ^[50]^ ^[ii-iv]^ using the OPLS 2005 force field ^[v]^. Simulation conditions were maintained at 1.0 atm constant pressure by the Nose´-Hoover Langevin piston method ^[vi-vii]^ and at 310 K constant temperature by Langevin dynamics with a damping coefficient at 0.5 ps. The time step used for the simulations was 1fs, and the coordinates were recorded every 20 ps. A cutoff of 12Å was used for short-range, nonbonded interaction, and long-range electrostatic interactions were computed using the particle mesh Ewald method. All simulations subject for (20000ps) 20nsec simulation time for α-syn and rotenone bound α-syn.

**Nuclear Magnetic Resonance Spectroscopy:** For ^15^N labeled α-syn, the E. coli cells (transformed with pT7-7 α-syn WT plasmid, a gift from Hilal Lashuel Lab ) were grown in the M9 minimal media containing ^15^NH4Cl(CIL, MA, USA) as the sole sources of nitrogen. The expression and purification of labelled α-syn was done as previously described. The labeled α-syn was aggregated for fibril formation at linear shaking (600rpm) for 48h at 37°C as described above. All the NMR spectra of α-syn monomers, fibrils and rotenone induced fibrils (molar ratios of α-syn:ROT;1:5) were recorded on 800 MHz Bruker Avance III FT-NMR spectrometer with a cryoprobe at 300K. Uniformly 15N labelled protein samples of α-syn (concentration ~150 µM) dissolved in 50 mM aqueous sodium phosphate buffer containing 10% (v/v) D2O, 300 mM NaCI and buffer pH 6.8 was used. Further, 1H-15N HSQC (hetero-nuclear single quantum correlation) pulse sequence was used to investigate the chemical shift perturbation and intensity alterations of monomeric α-syn (before/after fibrillation) in the presence and absence of rotenone. NMR spectral processing and analysis was done using software tools, TOPSPIN (v3.5) (http://www.bruker-blospin.com/nmr.html) and CARA respectively.

**a**

**b**

**c** O OR


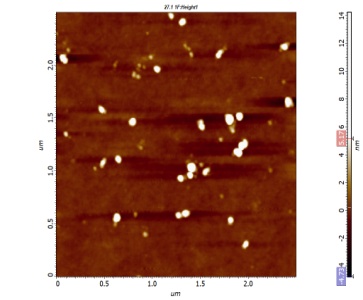

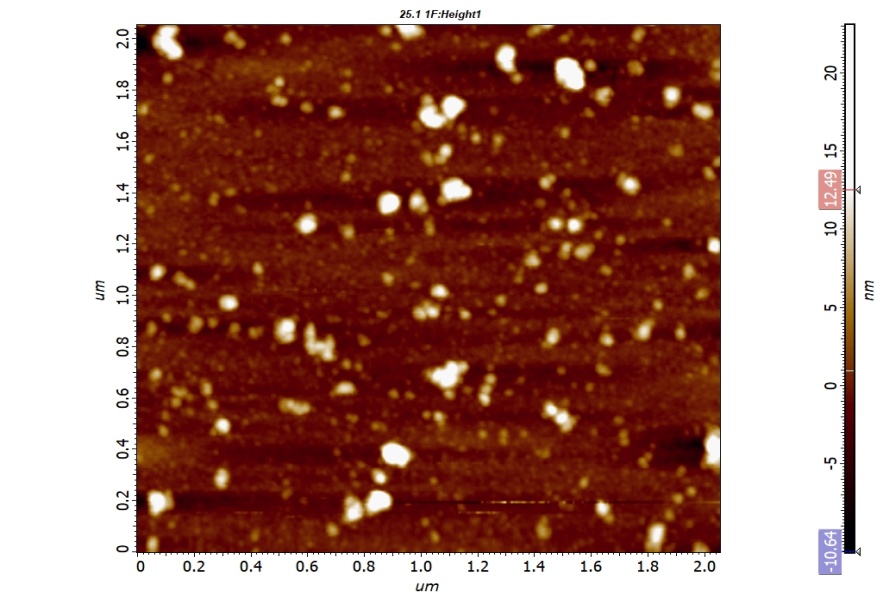


**Fig S1: Characterization of α-syn oligomers.** Dot blot based characterization of purified α-syn monomers(M), oligomers, fibrils (O,F) and rotenone-induced oligomers and fibrils (OR, FR) using monomers, oligomers(A11) and fibrils(syn211) specific antibodies (**a**) Molecular weight characterization of α-syn monomers, oligomers and rotenone induced oligomers by size exclusion chromatography (**b**). The morphology of the α-syn oligomers(O) and rotenone induced oligomers (OR) was monitored using AFM (**c**).

**Table S1: Comparative molecular weight analysis of different α-syn species formed on aggregation pathway.**

| α-syn Species | Hydrodynamic radii (nm) determined by DLS | Molecular weight Determined by size exclusion chromatography (KDa) |
| --- | --- | --- |
| Monomers (M) | 5.7 | 14 |
| Oligomer (O) | 52 | approx 500 |
| Rotenone induced Oligomer (OR) | 52 | approx 500 |
| Fibrils (F) | 855.2 | Not Detected |
| Rotenone induced Fibrils (FR) | 1152.4 - 5000 | Not Detected |

**Table S2: quantification of the secondary structure content in α-syn aggregated species**. The analysis of Far UV spectra by BestSel software (**a**) and K2D3 software (**b**) for α-syn oligomers (O) rotenone induced oligomers (OR) α-syn fibrils (F) and rotenone induced fibrils (FR) shows loss of α-helix content in rotenone induced aggregates.

**(a)**

| α-Syn species | α-helices  (%) | β sheets  (%) | Turns  (%) | Others  (%) |
| --- | --- | --- | --- | --- |
| Oligomers(O) | 12 | 31 | 14 | 43 |
| Rotenone induced Oligomers(OR) | 0 | 43.8 | 17.7 | 38.5 |
| Fibrils(F) | 8 | 36 | 14 | 42 |
| Rotenone induced fibrils (FR) | 0 | 46 | 13 | 41 |

**(b)**

| α-Syn species | α-helices  (%) | β sheets  (%) |
| --- | --- | --- |
| Oligomers(O) | 44.79% | \| 10.17% \| \| --- \| |
| Rotenone induced Oligomers (OR) | 4.41% | 38.24% |
| Fibrils(F) | 34.23% | 13.91% |
| Rotenone induced fibrils (FR) | 1.65% | 43.89% |


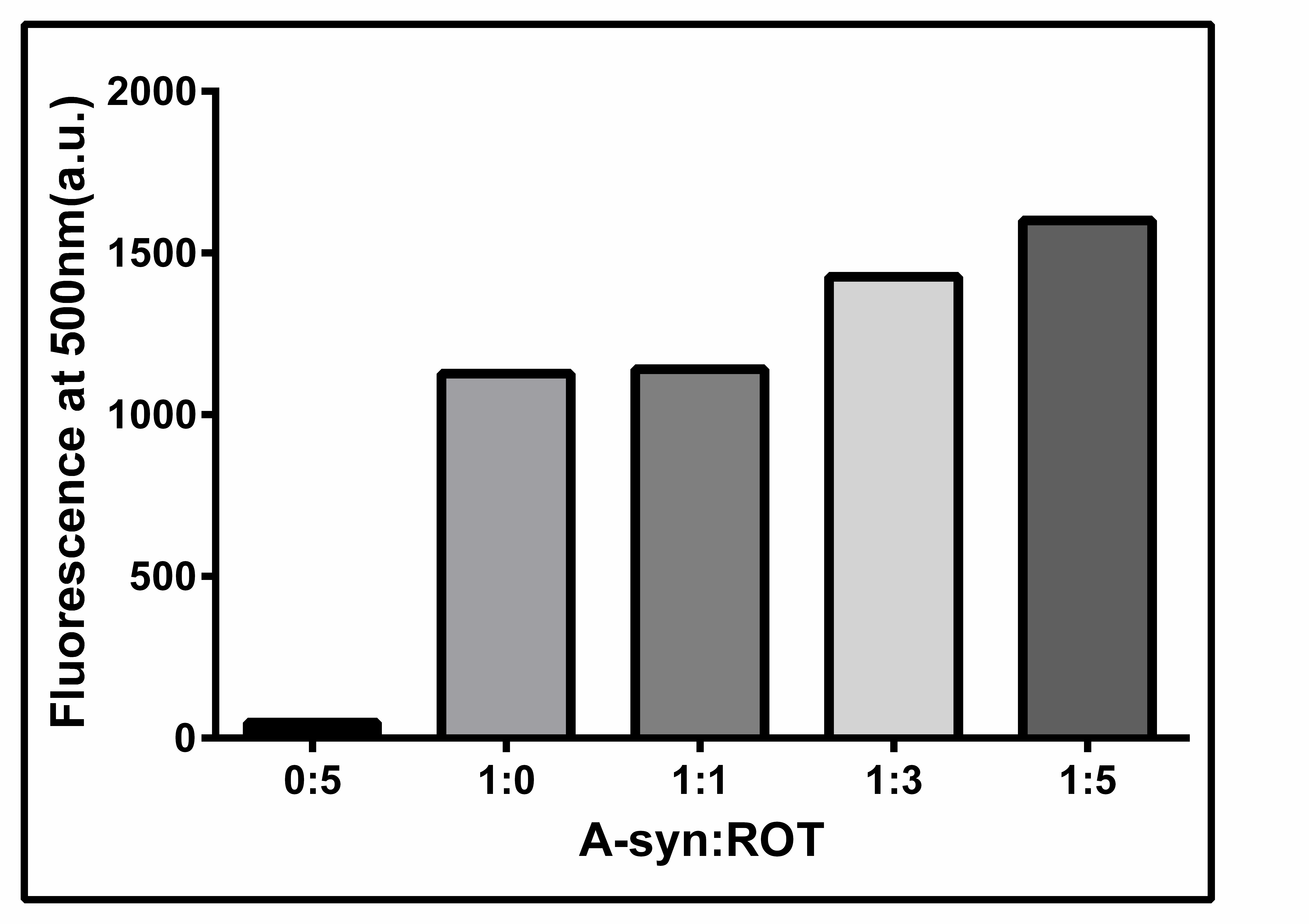


**Fig.S2: Rotenone influences the hydrophobicity of α-syn**. Comparative plot for change in hydrophobicity of α-syn by rotenone measured using fluorescence emission of Bis-ANS.


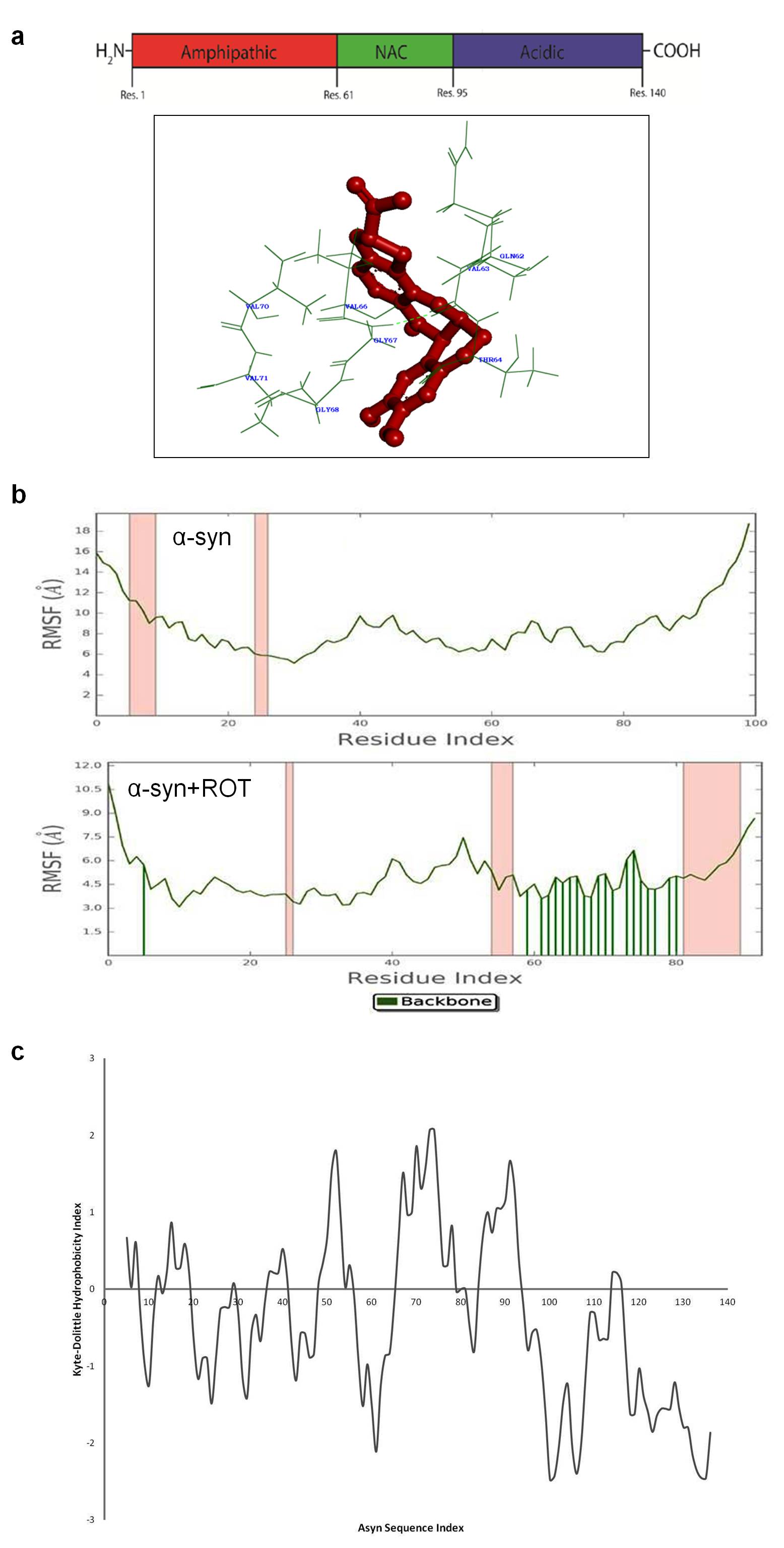


Continues to next page.

**
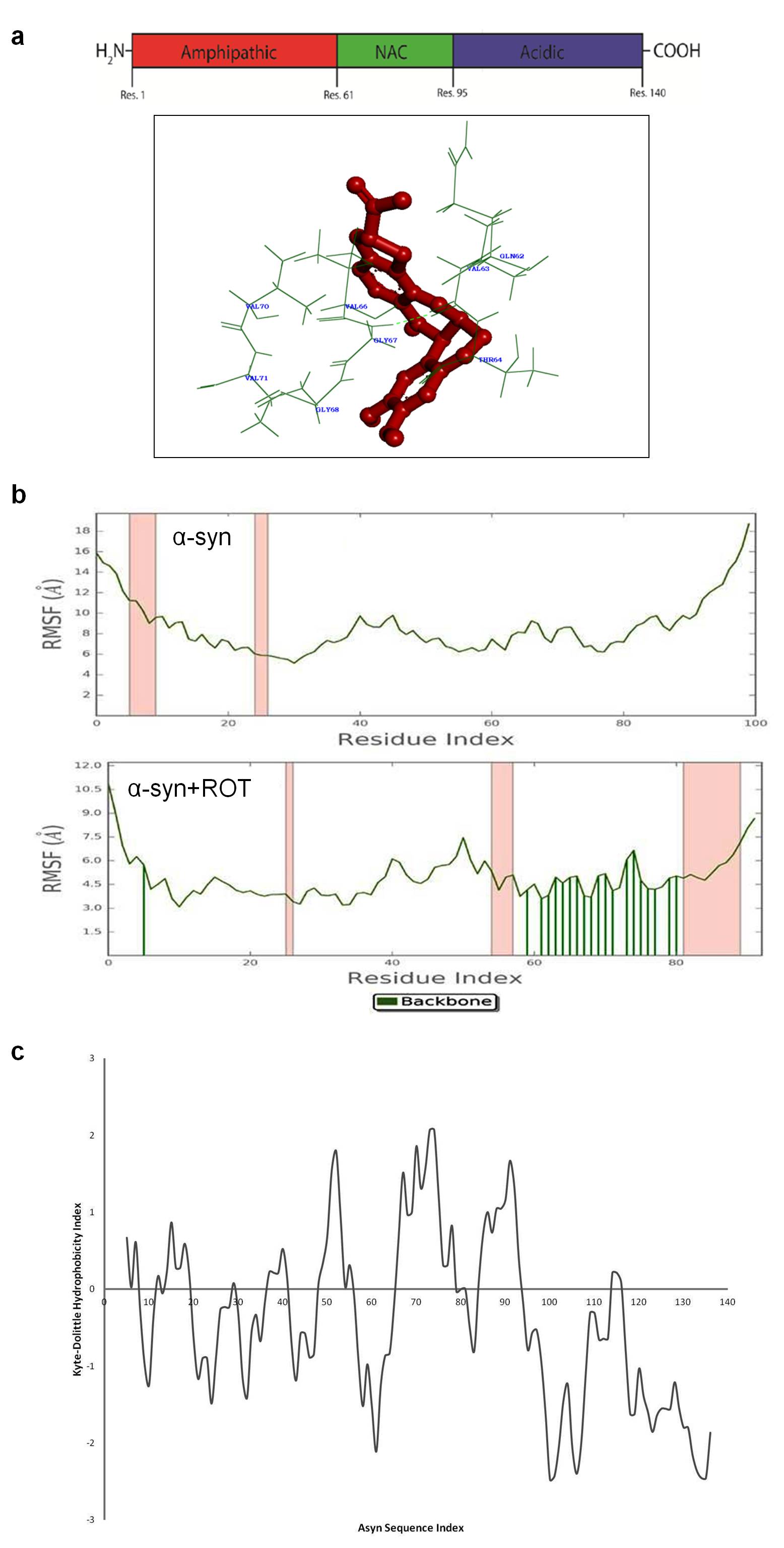
**

**Figure S3: Rotenone-α-syn binding and local contact mapping.** The representation of α-syn amino acid sequence to different regions and the 3D interaction diagram of the complex formed between the ligand and the protein **(a)**. The protein is represented in green, ligand in red and the interacting residues are coloured blue. The Root Mean Square Fluctuation (RMSF) for characterizing local changes along the protein chain **(b)**. The green straight lines indicate ligand interaction with protein in aggregation prone regions. The red bars indicate that these regions form α helix for more than 70 % of the time during simulation in the presence of rotenone as compared to α-syn alone. No β sheets are observed during the duration of run used in this study. Kyte-doolittle plot for α-syn hydrophobicity prediction at amino acid levels. **(c)**


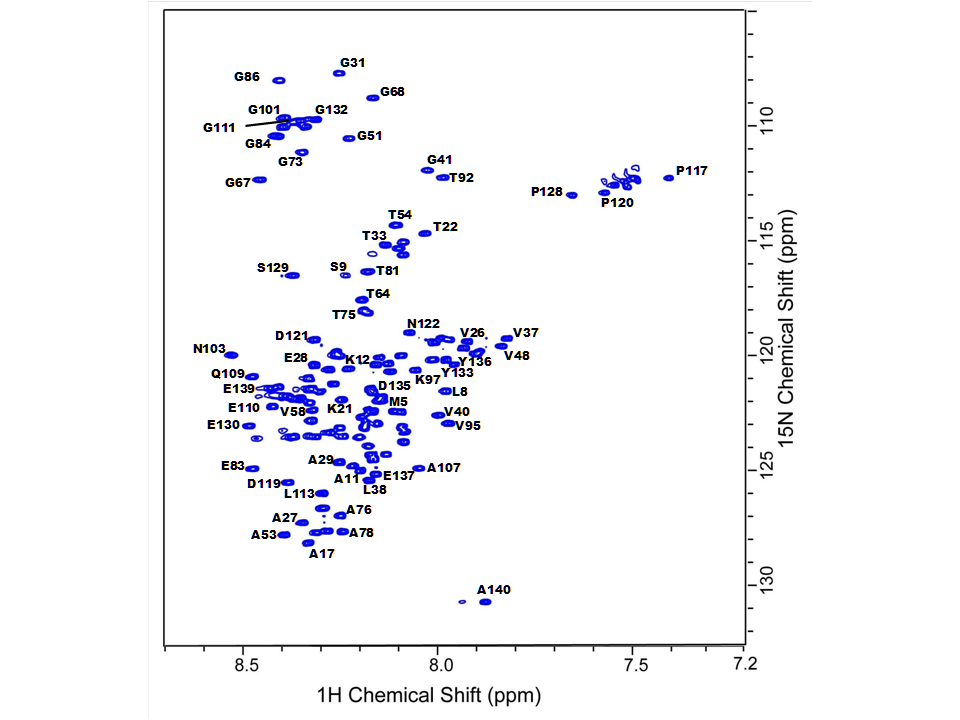


**Fig S4: 1H-15N HSQC spectrum of α-syn monomers** **as per earlier assignment** ^[36]^.

**(a)**

**
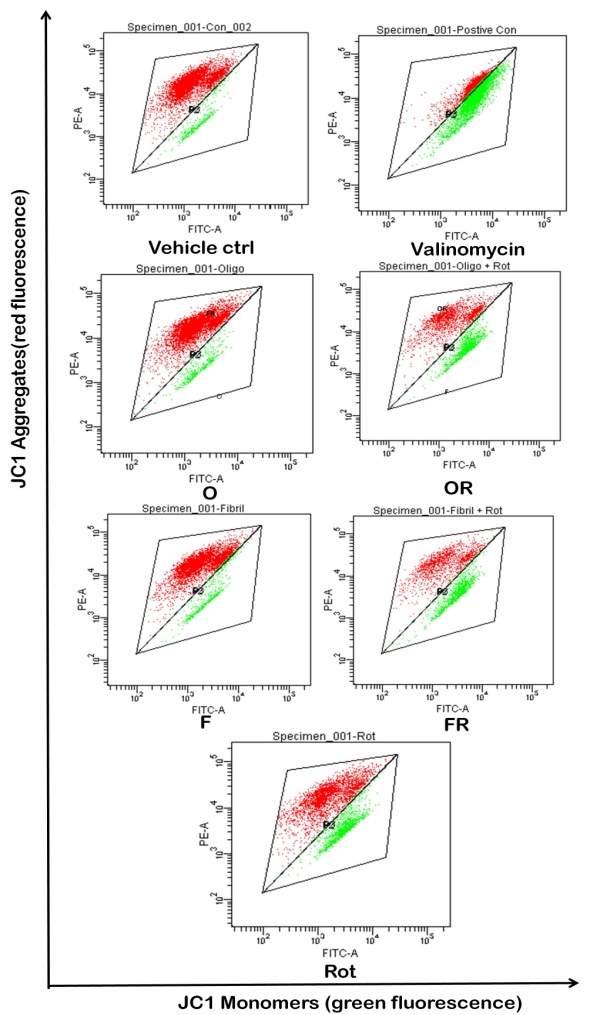
(b)**

**FigS5: Effect of α-syn oligomers and fibrils on cellular oxidative stress and membrane potential.**  α-syn oligomers and fibrils do not induce oxidative stress in SH-SY5Y cells. The cellular ROS generation assessed by detecting DCF fluorescence after 6h exposure of different concentrations(as indicated in graphs) of α-syn oligomers, fibrils (O, F) and rotenone induced α-syn oligomers an fibrils (OR, FR) (**a**). α-Syn induced a loss of mitochondrial membrane potential in SH-SY5Y cells (**b**). Effect of α-syn aggregated species combination after 24 h treatment duration on mitochondrial membrane potential in SH-SY5Y cells as measured by JC-1 staining. The figure represents one of the triplicate. Cells with rotenone or PBS alone were taken as positive or negative control in above experiment. Statistical significance was evaluated by One way ANOVA analysis data are expressed as means and +/- SE (n=3),nsP>0.05, *P<0.05; **P<0.01; ***P <0.001 .

**SI References:**

1. Holmqvist S, Chutna O, Bousset L, et al. Direct evidence of Parkinson pathology spread from the gastrointestinal tract to the brain in rats. Acta Neuropathol. 2014;128(6):805-820. doi:10.1007/s00401-014-1343-6
2. Kevin J. Bowers, Edmond Chow, Huafeng Xu, Ron O. Dror, Michael P. Eastwood, Brent A. Gregersen, John L. Klepeis, Istvan Kolossvary, Mark A. Moraes, Federico D. Sacerdoti, John K. Salmon, Yibing Shan, and David E. Shaw, "Scalable Algorithms for Molecular Dynamics Simulations on Commodity Clusters," [Proceedings of the ACM/IEEE Conference on Supercomputing (SC06), Tampa, Florida, 2006, November 11-17](http://dl.acm.org/citation.cfm?id=1188455).
3. Schrödinger Release 2017-1: Desmond Molecular Dynamics System, D. E. Shaw Research, New York, NY, 2017. Maestro-Desmond Interoperability Tools, Schrödinger, New York, NY, 2017.
4. Shivakumar, D.; Williams, J.; Wu, Y.; Damm, W.; Shelley, J.; Sherman, W., "Prediction of Absolute Solvation Free Energies using Molecular Dynamics Free Energy Perturbation and the OPLS Force Field," [J. Chem. Theory Comput., 2010, 6, 1509–1519](http://pubs.acs.org/doi/abs/10.1021/ct900587b)
5. Martyna, G. J., D. J. Tobias, and M. L. Klein. 1994. Constant pressure molecular dynamics algorithms. J. Chem. Phys. 101:4177–4189.
6. Feller, S. E., Y. H. Zhang, R. W. Pastor, and B. R. Brooks. 1995. Constant pressure molecular dynamics simulation — the Langevin piston method. J. Chem. Phys. 103:4613–4621.
7. Darden, T., D. York, and L. Pedersen. 1993. Particle mesh Ewald. An N_log(N) method for Ewald sums in large systems. J. Chem. Phys. 98:10089–10092.

*All numbered references are quoted and listed in main text file.
